# Evaluation of Doppler Effect Error Affecting the Radio Altimeter Altitude Measurements

**DOI:** 10.3390/s23010177

**Published:** 2022-12-24

**Authors:** Marek Češkovič, Pavol Kurdel, Natália Gecejová, Ján Labun, Miroslav Laššák

**Affiliations:** 1 Faculty of Aeronautics, Technical University of Košice, Rampová 7, 041 21 Košice, Slovakia; 2 Faculty of Electrical Engineering and Informatics, Technical University of Košice, Letná 9, 042 00 Košice, Slovakia; 3 Honeywell Flight Systems CoE, Tuřanka 100/1387, 627 00 Brno, Czech Republic

**Keywords:** doppler shift, dynamic altitude change, FMCW radio altimeter

## Abstract

The measurement of the real altitude of aircraft is usually done using an aviation radio altimeter (ALT). A radio altimeter provides crucial information about the instantaneous (radio) altitude of aircraft, helicopter, or unmanned aerial vehicle, to the pilot or another assistance system, such as an autopilot or an anti-collision system. However, this flight altitude measurement is affected by several errors, methodological errors and the operating frequency and modulation parameters instability, or the Doppler shift error. This article is focused on the evaluation of how the Doppler effect error develops during the operation of an ALT and its potential use as an information carrier concerning a possible loss of radio altitude, leading to dangerous situations. This paper briefly explains in a theoretical and practical way how this error develops and how it can affect the process of creation of height impulses. Practical experiments were conducted and evaluated in this research, and a theoretical design of a simple circuit capable of signalization of radio altitude loss presented. As the Doppler shift error was previously recognized solely as a measurement error, it could be used in a new function as a source of supplemental warning information.

## 1. Introduction

The altitude measurement during a flight is the most crucial information for achieving flight safety [1]. Altitude is usually measured by three different measurement principles, by measuring the change in static atmospheric pressure (barometric altimeter), by propagation and reflection of radio waves emitted towards the ground (radio altimeter) or the newest principle, by satellite navigation systems (GPS altitude) [2,3,4,5]. The radio altimeter is a device which has been used in aviation for instantaneous altitude measurement for almost eighty years [6,7]. These devices have developed and obtained the usual accuracy of ±30 cm. Information of the so-called real (actual) altitude above the terrain, or runway, is crucial for a lot of aircraft systems, like Auto Flight systems (Automatic Flight Guidance and Control Systems, Stick pusher/shaker, Flight Director, Thrust reversers, Autothrottle, Flight Controls, Flight Envelope Protection Systems), anticollision systems (Ground Proximity Warning Systems—GPWS, Traffic Collision Avoidance Systems—TCAS, Windshear detection, Tail strike prevention), and assistance aircraft systems (height above ground shown on Primary Flight Display, Take-off guidance systems, Engine and wing anti-icing systems) [5,6,7].

The FMCW (Frequency Modulated Constant Wave) radio altimeters are the most used radio altimeters in aviation [7,8]. They use the harmonic, high-frequency, frequency-modulated signal to measure the aircraft’s height above the earth’s surface. When compared to the other sources of actual flight altitude information, the radio altimeter is the most accurate one, and its information is used in many crucial aircraft systems [7,8]. The measured height is usually in the range of 0 m to 750 m—depending on the type of aircraft on which the radio altimeter is installed and used. Radio altimeters are mainly used at low flight altitudes and in the final stages of flight, usually during the most dangerous landing guidance and the landing process or in the hovering phase or approach of the helicopter [9,10].

Altitude measurements by radio altimeters are affected by several measurement inaccuracies, which were discussed in [11,12]. Some of these inaccuracies cannot be influenced and are fixed by the circuit design of the radio altimeter. However, others can be quantified and their overall effect on the accuracy of height measurements calculated [12,13]. One of the measurement errors, which cannot be altered, is the radio altimeter dynamic error. This error is constant (t ≈ 0.5 s) during all operations and is caused by the measurement method and its internal circuitry.

The present research focuses on the error of the radio altimeter with frequency modulation, which is created due to the influence of the Doppler effect during the aircraft’s descent or ascend. This error arises because when the aircraft is descending (or ascending), the value of the difference frequency, which carries information about the real flight height, changes by the value of the Doppler frequency [14,15]. This will result in a frequency shift of the evaluated signals, and the error of measuring the real (instantaneous) height measured with some radio altimeters may exceed the tolerated value [16,17,18,19,20]. This Doppler frequency value represents the increase in the frequency of the received signal over the transmitted signal if the aircraft is descending and the decrease in frequency if the aircraft is climbing. This phenomenon can be used to determine the prognosis of the aircraft/helicopter/unmanned aerial vehicle (UAV) radio altitude loss—the potential impact to the terrain. In the material and methods chapter, we describe the phenomenon of Doppler shift creation and how it affects the difference frequency creation, as it is the main principle of how the FMCW radio altimeter measures height. In the main section, the hypothesis of using the Doppler shift for predicting altitude loss is discussed together with an analysis of flight and laboratory experiments. The results are discussed in the fourth chapter.

## 2. Materials and Methods

**Figure 1:**
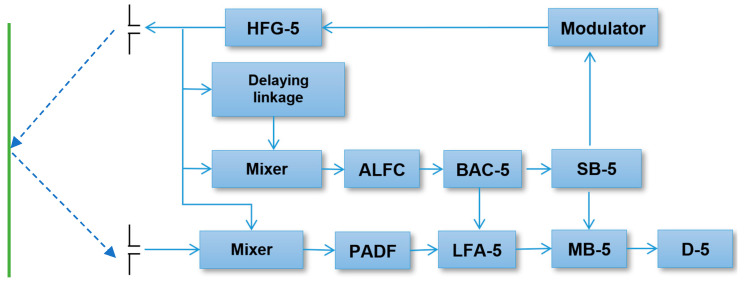
Block diagram of RV-5 type FMCW Radio altimeter, where HFG-5 is a high-frequency generator, ALFC is the low-frequency amplifier of a self-check signal, BAC-5 is the block for automatic adjusting of the radio altimeter constant, PADF is the preamplifier of a difference frequency, LFA-5 is the low-frequency amplifier, MB-5 is measurement block, SB-5 contains the self-check circuitry, and D-5 is the indicator.

The radio altimeter principle is that its transmitter (HFG-5 in Figure 1) produces a high-frequency, frequency-modulated signal u_v_(t), which is radiated towards the earth’s surface via the transmitting antenna.

**Figure 2:**
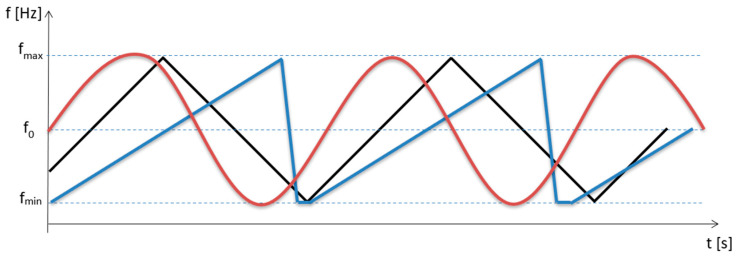
Radio altimeters use the three most common types of modulation signals. The sinusoidal (**red**), the symmetric sawtooth (**black**) and the asymmetric sawtooth (**blue**) instantaneous frequencies are shown.

**Figure 3:**
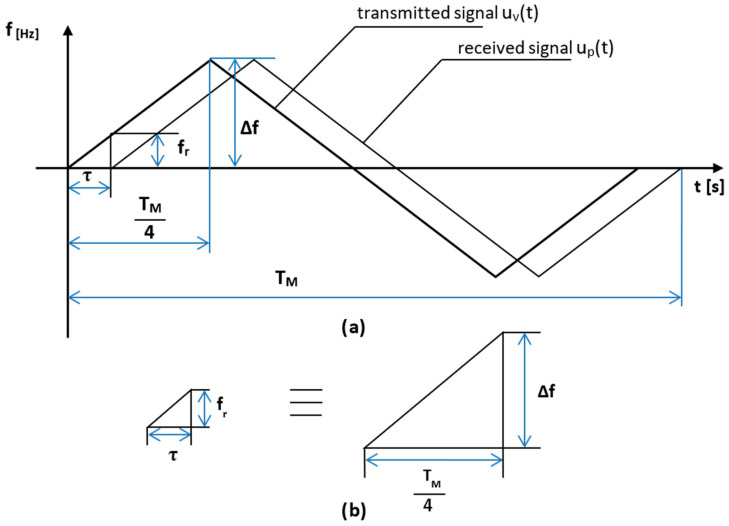
The creation of difference frequency *f_r_*. Courses of transmitted and received radio altimeter signal (**a**), together with individual parameters like modulation period and time delay, are also shown. On (**b**) the triangles for determining the fundamental equation of the FMCW radio altimeter are shown.

One of the three most common functions is used as the modulation frequency (the waveforms of which are shown in Figure 2) [7]. In our case, the radio altimeter uses a symmetric sawtooth modulating signal. As a result, the course of the signal reflected from the ground is the same as the course of the transmitted signal (Figure 3a), only it is delayed by the time required for the signal to propagate to the ground and back, which is expressed by Equation (1):

(1) $$\tau =\frac{2H}{c}$$

where:

*τ*—is the time delay of the received signal,

*H*—is the flight altitude of the aircraft,

*c*—is the propagation speed of electromagnetic waves.

We use a radio altimeter with a symmetric sawtooth modulation signal course for this research.

The reflected signal from the earth’s surface *u_p_*(*t*) and received signal by the receiving antenna is fed into a balanced mixer, where the direct signal *u_v_*(*t*) from the transmitter is fed simultaneously. As a result of the reflected signal being time-delayed, the frequency of the direct signal at any instant is different from the frequency of the reflected signal.

At the output of the balanced mixer, after the separation of unwanted components, a differential signal *u_r_*(*t*) arises, whose frequency *f_r_* is expressed by Equation (2):

(2) $$f_{r}\left(t\right)=\left|f_{v}\left(t\right)-f_{p}\left(t\right)\right|$$

where:

*f_r_*—is the difference frequency,

*f_v_*(*t*)—is the transmitted frequency,

*f_p_*(*t*)—is the frequency of the received signal.

The absolute value is applied in Equation (2) for a physical reason—the frequency is always positive.

It can be seen from the time courses (Figure 3a) that the size of the difference frequency, which depends on the time delay of the reflected signal, is constant for the most significant part of the modulation period, with the amplitude not changing and can be expressed by the following Equation (3):

(3) $$f_{r}=\frac{8\Delta ff_{M}}{c}H$$

where:

*f_r_*—is the difference frequency,

Δ*f*—is the frequency lift,

*f_M_*—is the modulation frequency,

*H*—is the flight altitude of the aircraft,

*c*—is the propagation speed of electromagnetic waves.

The time period *τ*, in which the difference frequency *f_r_* is not constant, is called the phase rotation band—at the middle point of this band, the function *f_r_*(*t*) is equal to zero.

Equation (3) is the fundamental equation of a radio altimeter with frequency modulation and constant modulation period. This equation is valid only in cases when it is possible to neglect the influence of the band rotation of the phase of the difference signal *u_r_*(*t*) and if we can consider that relationship (4) will be valid for the entire duration of the modulation period.

(4) $$f_{r}=constant$$

This condition is met only at low altitudes (approximately 2000 m). Due to this, these radio altimeters are named low-altitude radio altimeters [21,22].

### 2.1. Generation of (Height) Impulses in a Radio Altimeter with Frequency Modulation

The signal *u_r_*(*t*) from the balanced mixer is amplified by a difference signal amplifier and fed to the evaluation circuit, where the magnitude of the difference frequency is evaluated, corresponding to the aircraft’s actual flight height.

The basic equation of a radio altimeter with frequency modulation and a constant modulation period (3) is derived from the course of the modulation period while using the similarity of triangles (Figure 3b) representing the course of change in the value of the frequency of the transmitted signal over time.

If it is true that ΔABC ≡ ΔDEF, then the Equation (5) is:

(5) $$\frac{f_{r}}{\tau }=\frac{\Delta f}{\frac{T_{M}}{4}}=\frac{4\Delta f}{T_{M}}$$

where:

*f_r_*—is the difference frequency,

*τ*—is the time delay of the received signal,

Δ*f*—is the frequency lift,

*T_M_*—is the modulation period.

When Equation (1) applies, and at the same time, then Equation (6) expresses the value of the modulation period (TM):

(6) $$T_{M}=\frac{1}{f_{M}}$$

where:

*T_M_*—is the modulation period,

*f_M_*—is the modulation frequency.

At the same time, the equation is (7):

(7) $$\frac{cf_{r}}{2H}=4\Delta ff_{M}$$

*f_r_*—is the difference frequency,

*c*—is the propagation speed of electromagnetic waves,

Δ*f*—is the frequency lift,

*H*—is the flight altitude of the aircraft,

*f_M_*—is the modulation frequency.

The final form of the fundamental equation of the radio altimeter can be derived from these relationships (3)–(7).

**Figure 4:**
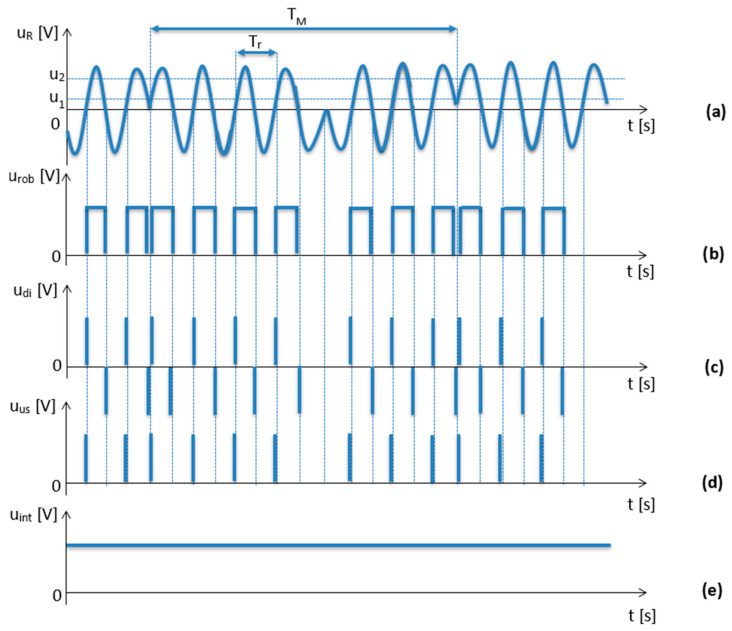
The time courses of signals as they are processed by the evaluation circuit of the radio FMCW altimeter. On (**a**), there is the harmonic signal of a difference frequency; in (**b**), there is the same signal after the shaping process. On (**c**), there is the detection process of the leading edges of the signal, and on (**d**), there is the signal after filtration of the negative impulses. Finally, on the (**e**) is the signal after integration.

The current radio altimeters use frequency meters as evaluation circuits, which are implemented as spectrum analyzers or as analogue or digital meters with direct or indirect differential frequency measurement. In terms of simplicity of operation and reliability, evaluation circuits with direct measurement of the difference frequency are most often used. The principle of their function consists of counting impulses, which are shaped from the difference signal u_r_(t) throughout the duration of the modulation period T_M_ (Figure 4).

As can be seen in Figure 4, the low-frequency difference signal u_r_(t) is fed to a two-way limiter after filtering and necessary amplification. Double-sided constraints provide greater fault tolerance. The limit thresholds u_1_ and u_2_ are usually chosen, as shown in Figure 4a. The two-way limited signal in the form of voltage impulses u_rob_(t) (Figure 4b) proceeds to the derivative circuit. The voltage peaks (Figure 4c) that we get at its output go to the DC rectifier to filter negative voltage peaks. As a result, the positive derivative impulses u_di_(t) (Figure 4d) have the same frequency as the difference signal frequency.

The obtained voltage impulses feed either a classical integrator, as is the case with older types of radio altimeters with analogue output, or a decade counter, which is used in more modern radio altimeters. The output is a DC voltage u_int_(t) (Figure 4e), which is proportional to the number of voltage impulses.

In addition to the mentioned circuit, which forms the height measurement channel, the radio altimeter also contains other auxiliary circuits, such as the circuit for signaling the selected value of the dangerous height, the radio altimeter status self-checking circuit, the radio altimeter testing circuit, and more. However, these circuits do not affect the height measurement itself and, for this reason, will not be discussed further.

### 2.2. Doppler Effect in Radio Altimeters with Frequency Modulation

In this subsection, the problem of radio altimeter error with frequency modulation, created by the influence of the Doppler effect during the aircraft’s descent in landing mode, is briefly discussed. When the aircraft/helicopter/UAV is descending, the value of the difference frequency, which carries information about the height, changes by the value of the Doppler frequency. This will result in a frequency shift of the evaluated signals. In the case of an increase in the frequency of the received signal compared to the transmitted signal, the aircraft is descending. In the opposite case, i.e., when the frequency of the received signal decreases compared to the transmitted signal, the aircraft is climbing.

To explain the frequency shift due to the Doppler effect of the radio altimeter transmitter signal during its reception, we assume that the aircraft descends at a vertical speed vv. The electromagnetic wave of the radio altimeter transmitter signal is emitted with a frequency *f_v_*(*t*) and falls on the earth’s surface with a frequency shift $f_{v}^{1}$ (t), which is expressed by Equation (7):

(8) $$f_{v}^{1}\left(t\right)=f_{v}\left(t\right)\frac{c}{c-v_{v}}$$

where:

*f_v_*(*t*)—is the transmitted frequency,

$f_{v}^{1}$(*t*)—is the transmitted frequency, impacting on the earth’s surface, with a frequency shift,

*C*—is the propagation speed of electromagnetic waves,

*v_v_*—is the vertical speed of the aircraft.

Assume that the signal source is moving towards the ground and the observer is standing stationary on the ground. The signal is emitted perpendicular to the earth’s surface, and the vertical component of the aircraft’s speed during descent corresponds to the radial component of the propagating signal—this can be assumed due to the very low speed of the aircraft compared to the speed of propagation of electromagnetic waves. This fact is influenced by the layout of the antennas on the fuselage.

After the signal reflects off the earth’s surface, the earth becomes the secondary source of the signal, and the receiving antenna represents the observer. As the aircraft descends, this corresponds to the case where the observer approaches the source, and the received frequency is given by the Equation (9):

(9) $$f_{v}^{2}\left(t\right)=f_{v}^{1}\left(t\right)\frac{c+v_{v}\;}{c}$$

where:

$f_{v}^{2}$(*t*)—is the received frequency,

$f_{v}^{1}$(*t*)—is the transmitted frequency, falling on the earth’s surface, with a frequency shift,

*c*—is the propagation speed of electromagnetic waves,

*v_v_*—is the vertical speed of the aircraft.

Modifying Equation (9) by adding from Equation (8), results in Equation (10):

(10) $$f_{v}^{2}\left(t\right)=f_{v}\left(t\right)\frac{c}{c-v_{v}}\frac{c+v_{v}}{c}=f_{v}\left(t\right)\frac{c+v_{v}}{c-v_{v}}$$

where:

$f_{v}^{2}$(*t*)—is the received frequency,

*f_v_*(*t*)—is the transmitted frequency,

*c*—is the propagation speed of electromagnetic waves,

*v_v_*—is the vertical speed of the aircraft.

This expression (10) represents the Doppler effect. Therefore, the difference between the frequency of the received and transmitted signals is the Doppler frequency *f_D_*, which can be expressed as Equation (11):

(11) $$f_{D}=f_{v}^{2}\left(t\right)-f_{v}\left(t\right)=f_{v}\left(t\right)\frac{c+v_{v}}{c-v_{v}}-f_{v}\left(t\right)=f_{v}\left(t\right)\left(\frac{c+v_{v}}{c-v_{v}}-1\right)\;=f_{v}\left(t\right)\left(\frac{c+v_{v}-c+v_{v}}{c-v_{v}}\right)=f_{v}\left(t\right)\frac{2v_{v}}{c-v_{v}}$$

where:

*f_D_*—is the Doppler frequency,

*f_v_*(*t*)—is the transmitted frequency,

$f_{v}^{2}$(*t*)—is the received frequency,

*c*—is the propagation speed of electromagnetic waves,

*v_v_*—is the vertical speed of the aircraft.

Assuming that *v_v_* << *c*, Equation (11) can be written simply as Equation (12):

(12) $$f_{D}=f_{v}\left(t\right)\frac{2v_{v}}{c}$$

where:

*f_D_*—is the Doppler frequency,

*f_v_*(*t*)—is the transmitted frequency,

*c*—is the propagation speed of electromagnetic waves,

*v_v_*—is the vertical speed of the aircraft.

**Figure 5:**
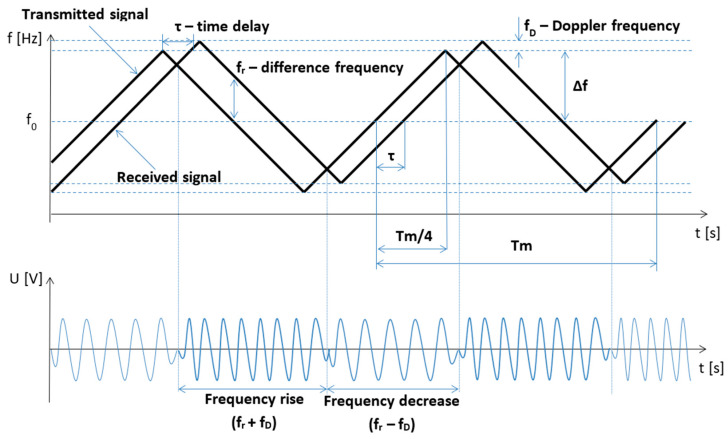
Illustration of Doppler frequency shift affecting course of radio altimeter received signal.

This Doppler frequency value represents the increase in the transmitted signal frequency if the aircraft is descending and the decrease in signal if the aircraft is climbing (Figure 5). For simplicity, we set (13):

(13) $$f_{v}\left(t\right)=f_{0}$$

where:

*f_v_*(*t*)—is the transmitted frequency,

*f*_0_—is the middle frequency of the carrier signal.

In such a case, a difference in frequency arises with the inclusion of the Doppler frequency, which is expressed by the Equation (14):

(14) $$f_{rD}=f_{r}\pm \;f_{D}=\frac{8\Delta ff_{M}}{c}H\pm \;f_{0}\frac{2v_{v}}{c}$$

where:

*f_rD_*—is the difference frequency, including the Doppler frequency,

*f_r_*—is the difference frequency,

*f_D_*—is the Doppler frequency,

*f_M_*—is the modulation frequency,

*c*—is the propagation speed of electromagnetic waves,

Δ*f*—is the frequency lift,

*H*—is the flight altitude of the aircraft,

*v_v_*—is the vertical speed of the aircraft,

*f*_0_—is the middle frequency of the carrier signal.

Figure 5 shows that if the frequency modulation has the shape of a symmetrical saw, then in one half-period of the modulation frequency, the difference frequency increases due to the Doppler frequency and decreases in the other half-period. The lower panel in Figure 5 shows that during modulation with a symmetrical saw, the negative influence of the Doppler frequency does not manifest itself because the mean value of the difference frequency does not change during one modulation period. It will not be reflected in altimeters that use a symmetrical saw in modulation—for example, the altimeters RV-5, RV-15, KRA-405, or RV-UM. Altitude measurement by radio altimeters is generally affected by several measurement inaccuracies (methodological error, instability error of frequency modulation parameters, dynamic error, instrument error, Doppler effect error, radio altimeter parameter fluctuation error, displacement error, error due to external conditions and parasitic modulation error). The total measurement error of the radio altimeter we were examining (RV-5) is ± 0.75 m (which represents 100% inaccuracy). The methodological error has the largest share in this inaccuracy (70%); frequency modulation parameter instability error is next (20%); an aircraft/helicopter flight dynamics error of 6% and remaining measurement errors such as parasitic modulation or Doppler shift error represent the remaining 4%. Data for such a statistical evaluation were obtained through many years of research by the authors in the field of radio altimeters [11,12,13,14].

## 3. Potential Use of the Doppler Effect for Prediction of the Altitude Loss

**Figure 6:**
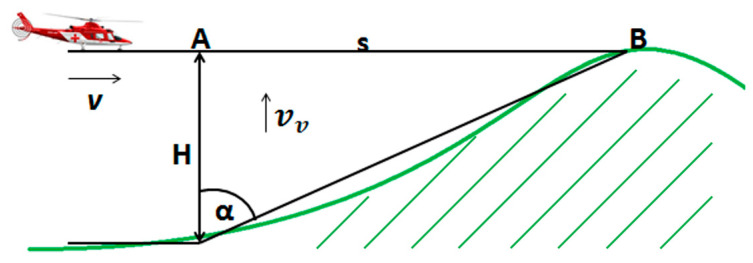
Illustration of scenario where the aircraft during level flight at point A starts to approach the hill, where B is the point, where flight altitude H will be zero.

In this hypothesis, let us assume that the aircraft/helicopter/UAV, equipped with the FMCW radio altimeter, with a symmetric sawtooth modulation signal, constantly flies on one flight level at a height H above the terrain. Furthermore, let us also assume that the terrain begins to rise towards the flying aircraft from a certain point A (Figure 6).

The loss of the aircraft altitude occurs at point *B*, at time *t* when the aircraft passes the flight path *s*, so (Equation (15)):

(15) $$t=\frac{s}{v}=\frac{H\tan\alpha }{v}$$

where:

*t*—is the time to the potential collision with the terrain,

*s*—is the flight path of the aircraft,

*v*—is the flight-speed of the aircraft,

*α*—is the angle of attack of the terrain.

When the terrain is coming up to the aircraft, the Doppler frequency (*f_D_*) is shaped, which is proportional to the vertical component of the velocity *v_v_*. According to Figure 5, the vertical component of the velocity is determined by Equation (16):

(16) $$v_{v}=\frac{v}{\tan\alpha }$$

where:

*v_v_*—is the vertical speed of the aircraft,

*v*—is the flight-speed of the aircraft,

*α*—is the angle of attack of the terrain.

Substituting Equation (16) into Equation (15), results in Equation (17) for determining the time, which represents the ratio between the flight height of the aircraft and the vertical component of the velocity:

(17) $$t=\frac{H}{v_{v}}$$

where:

*t*—is the time to the potential collision with the terrain,

*H*—is the flight altitude of the aircraft,

*v_v_*—is the vertical speed of the aircraft.

The flight height of the aircraft H is evaluated by a conventional radio altimeter and given by the difference frequency *f_r_* value. A radio altimeter converts the measured value of the difference frequency *f_r_* proportional to height into a voltage *U_H_* proportional to height.

The vertical component of the velocity *v_v_* corresponds to the value of the Doppler frequency *f_D_*. To determine the dangerous time, it is necessary to convert the value of the Doppler frequency *f_D_*, proportional to the vertical component of the velocity, into the voltage *U_D_*, which is proportional to the vertical component of the velocity. The state when these two voltages reach a certain ratio determines the dangerous time that remains until the aircraft collides with the terrain. It is expressed by Equation (18):

(18) $$t=\frac{H}{v_{v}}=\frac{f_{r}}{f_{D}}=\frac{U_{H}}{U_{D}}$$

where:

*t*—is the time to the potential collision with the terrain,

*H*—is the flight altitude of the aircraft,

*v_v_*—is the vertical speed of the aircraft,

*f_r_*—is the difference frequency,

*f_D_*—is the Doppler frequency,

*U_H_*—is the voltage proportional to the flight altitude of the aircraft,

*U_D_*—is the voltage proportional to the vertical component of the aircraft’s flight-speed.

This hypothesis was patented by the authors and creates the theoretical background for using the former Doppler shift error, which affects the measurement of radio altitude by the FMCW radio altimeter, as a source of information for potential altitude loss leading to dangerous flight scenarios [23]. To get an answer to this hypothesis, the authors’ team conducted a series of experiments, which are discussed in the following sections and subsections.

### 3.1. Flight Experiment

**Figure 7:**
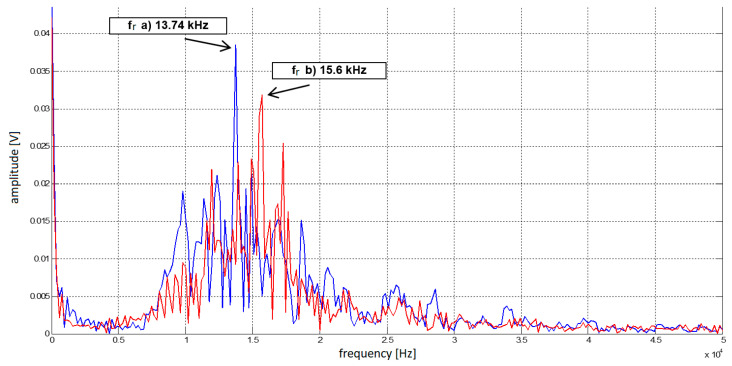
Spectral analysis of signals measured during the flight experiment showed that we had successfully recorded the presence of Doppler shift frequency in different frequency signals of the radio altimeter during a 3.8 s period flying towards the obstacle (hill) [14].

The real flight experiment with a similar scenario, as shown in Figure 6, was conducted to assess if the initial hypothesis was correct. The authors wanted to confirm that the emergence of the Doppler frequency increment to the difference frequency will occur and could be successfully evaluated [14]. For understandable reasons, the helicopter flew by at a set height just above the top of the hill. A recording device was connected to the radio altimeter, recording the difference frequency course during the experiment. Spectral analysis of the recorded difference signal proved that the frequency proportional to the flight height also contains the Doppler frequency—Figure 7 (15600 Hz –13740 Hz =1860 Hz; $f_{d}=\pm \;930\;\mathrm{Hz}$). Based on the flight parameters, the Doppler frequency can be calculated and compared with the data obtained from the spectral analysis of the difference signal. To determine the value of the Doppler frequency *f_D_*, it is, therefore, necessary to determine the value of the vertical component of the velocity *v_v_*.

The helicopter flight-speed during the experiment was 150 kmh^−1^. The value corresponds to the horizontal component of the velocity v = 41.67 ms^−1^. Then, using the function tan of the known value of the angle β and the horizontal component of the velocity v, the vertical component of the velocity *v_v_* can be calculated (Equations (19) and (20)).

(19) tanβ=tan36.5=0.74

(20) $$\tan\beta =\frac{v_{v}}{v}\to v_{v}=\tan\beta v=0.74\times 41.67=30.84\;{\mathrm{ms}}^{-1}$$

where:

*v_v_*—is the vertical speed of the aircraft,

*v*—is the flight-speed of the aircraft,

*β*—is the steepness of the hill.

The calculated value of the vertical component of the velocity is *v_v_* = 30.84 m·s^–1^. By introducing the value into Equation (20), it is possible to calculate the Doppler frequency *f_D_*. At the same time, the value of the carrier frequency of the RV-5 radio altimeter on which the experiment was performed is ($4.4\times 10^{9}$) Hz.

(21) $$f_{D}=\pm f_{0}\frac{v_{v}}{c}=\pm \left(4.4\times 10^{9}\right)\frac{2\times 30.84}{\left(3\times 10^{8}\right)}\dot{=}\pm 905\;\mathrm{Hz}$$

where:

*f_D_*—is the Doppler frequency,

*f*_0_—is the middle frequency of the carrier signal,

*v_v_*—is the vertical speed of the aircraft,

*c*—is the propagation speed of electromagnetic waves.

The calculated value of the Doppler frequency (*f_D_*) of 905 Hz is practically identical to the measured value of the Doppler frequency (*f_D_*) of 930 Hz and the difference is due to possible inaccuracies in calculations and errors during recording and the measurement itself.

This flight experiment showed us that the Doppler effect causing the Doppler frequency shift is measurable and can be evaluated [14]. This success led us to try to reproduce the flight experiment in laboratory conditions and to design a simpler way to detect and measure the Doppler effect of the radio altimeter by the microcontroller.

### 3.2. Simulation of Dynamic Height Change

The initial measurement (using the depolarization panel through electromagnetic waves) of discrete height values in indoor laboratory conditions with the RV-5 radio altimeter was successful and well documented in [11,12]. Subsequently, they also proved to be successful measurements of dynamic height change in indoor laboratory conditions in the same assembly of the measuring chain [11,12]. The success of the measurement relates to a new method, using a patented depolarization panel, through which the reflections of electromagnetic waves from surrounding obstacles in the interior laboratory environment were significantly suppressed. The only disadvantage of this dynamic height change was the low speed of movement that could be achieved in the laboratory’s interior. Therefore, the laboratory measurements were moved from the interior to the exterior, where it was planned to use a car’s movement for the simulation of a dynamic change in height. In this type of measurement, it was assumed that if the parasitic reflections of the radio altimeter transmitted high frequency (HF) carrier signal were eliminated in the interior using the depolarization panel, the parasitic reflections in the exterior would also be eliminated (which was also confirmed in the exterior measurements) [11,24,25].

**Figure 8:**
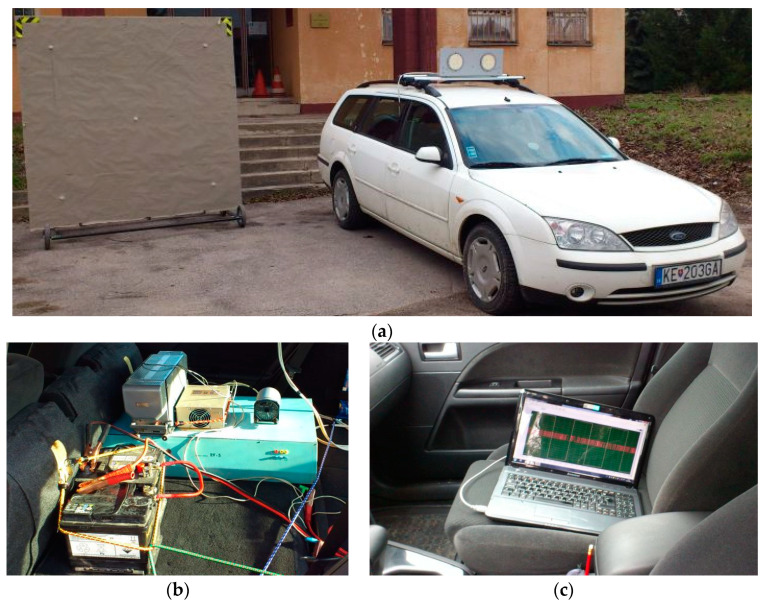
Setup for dynamic altitude change consisted of the depolarization panel as a reflective surface (simulating ground) together with an antenna system mounted on the roof of the car—(**a**), a computer with a LabView control panel for both visualization and recording of measured signals—(**b**) and RV-5 type FMCW radio altimeter stand mounted inside the car—(**c**).

We proceeded to the installation of a radio altimeter antenna system on the car (Figure 8a) and the radio altimeter stand was installed into the interior together with all necessary equipment, like DC and AC power sources and computer with respective programs (Figure 8b,c). The car’s speed was provided by its speed sensor, and the actual value was read through the OBD diagnostics port. Signals, which were measured and thus crucial for this evaluation, were the modulation signal and the difference frequency signal. They were measured by a National Instruments USB DAQ 6216 device. Signals were picked up from SB-5 (self-check control block for modulation signal) and MB-5 (measurement block for difference signal).

**Figure 9:**
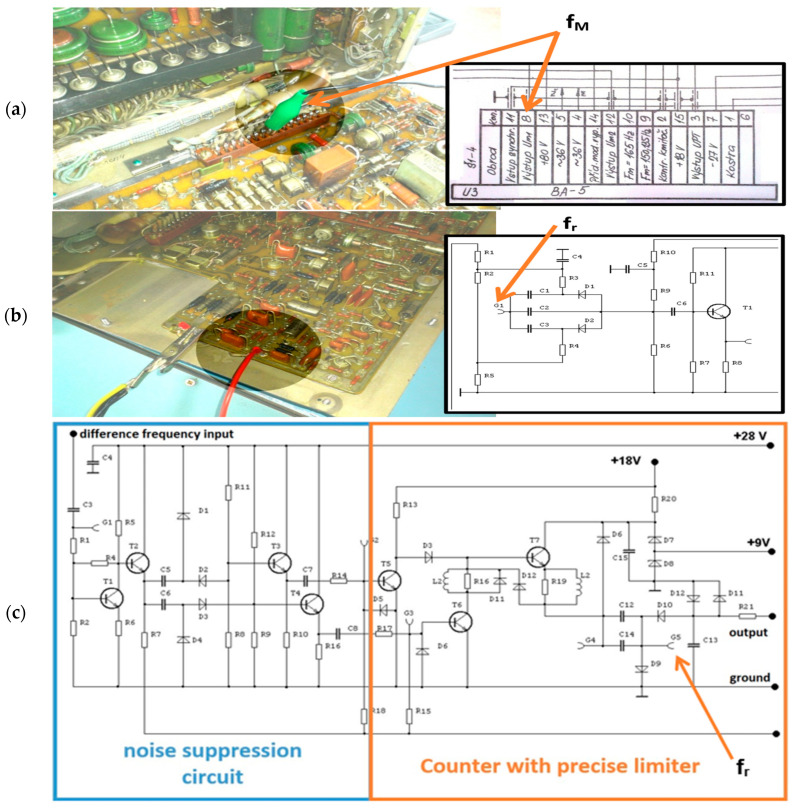
The point in the RV-5 circuitry from where the modulation signal *f_M_* (**a**) and difference signal *f_r_* (**b**) output was connected to the data acquisition device NI USB DAQ. The final point for picking up the difference frequency signal (after filtration) is shown on diagram (**c**).

At first, we measured the difference frequency signal *f_r_* at the output of the preamplifier of difference frequency signal (Figure 9b). However, the signal was in its “raw” form, affected by different types of noises and the shape of the respective impulses was insufficient. Therefore, we decided to use the radio altimeter’s own filtering and shaping circuits, and we connected them to the output where the signals are conditioned (Figure 9c).

The RV-5 type radio altimeter, which is the object of our research, uses several approaches to filter unwanted influences and stabilize the output information about the radio height. The first approach is the presence of so-called bandpass and bandstop filters, which create a total of four frequency characteristics. The RV-5 radio altimeter works with a frequency-modulated standing wave. A difference frequency is created by comparing the direct and reflected signal in the mixer, which can take values from 0 kHz to 150 kHz. The range of 0 kHz to 150 kHz corresponds to the range of 0 m to 750 m in height. To avoid the effect of coupling between antennas, intermodulations and multiple reflections from the ground, the following filters were used, which created a total of five frequency characteristics of the radio altimeter:

- In the range of very low altitudes (0–25 m), the first bandpass filter works 0–5 kHz, which filters all higher frequencies above 5 kHz.
- In the range of low heights (25–50 m), a second bandpass filter works, which filters frequencies up to 5 kHz and above 10 kHz.
- In the range of medium heights (50–150 m), a third bandpass filter works, which filters frequencies up to 10 kHz and above 30 kHz.
- In the range of high altitudes (150–250 m), a fourth bandpass filter works, which filters frequencies up to 30 kHz and from 50 kHz.
- In the range of very high altitudes (250–750 m), a fifth bandpass filter works, which filters frequencies up to 50 kHz and above 160 kHz.

High-frequency and low-frequency filters were also included in the circuits of the radio altimeter, specifically in the circuit of the differential frequency amplifier, together with circuits for switching frequency characteristics.

In the measurement block, there is a noise separator circuit (Figure 9c), the task of which is to shape the high impulses in such a way that during their subsequent integration into a direct current voltage, there is no erroneous evaluation of the impulses arising during the rotation of the phase of the high-frequency signal. Because measuring and evaluating only the Doppler frequency increase/decrease is complex and requires very precise timing, we decided to try a different approach. As part of testing the hypothesis and method, we decided to work with the evaluation of the difference in the number of impulses within the positive and negative part of one modulation period, which is reported as an error in height measurement due to the Doppler effect and causes the mentioned difference in the number of impulses. Thanks to the filters and the internal circuit solution of the radio altimeter, which has a stabilized modulation frequency, transmitter frequency, as well as circuits for automatic control and constant fine-tuning of the so-called constant of the radio altimeter, we could always rely on the entire number of height impulses taken from the radio altimeter measurement block.

With the radio altimeter mounted on the car, it was possible to simulate the dynamic change in the height of both types—the imaginary rise or descent of the aircraft/helicopter/UAV by approaching or retreating to the depolarization panel. In this way, it was possible to observe the emergence of a positive and a negative Doppler increase/decrease in frequency.

According to the hypothesis and mathematics in previous sections, the Doppler effect in radio altimeter circuits causes a frequency shift of the differential signal (Figure 5 and Figure 7). A positive or negative increment of the frequency difference makes it possible to determine whether the aircraft/helicopter/UAV is moving towards the earth’s surface—descending, or on the contrary, whether it is moving away—climbing. The magnitude of the frequency shift refers to the vertical component of the speed of this climb or descent.

**Figure 10:**
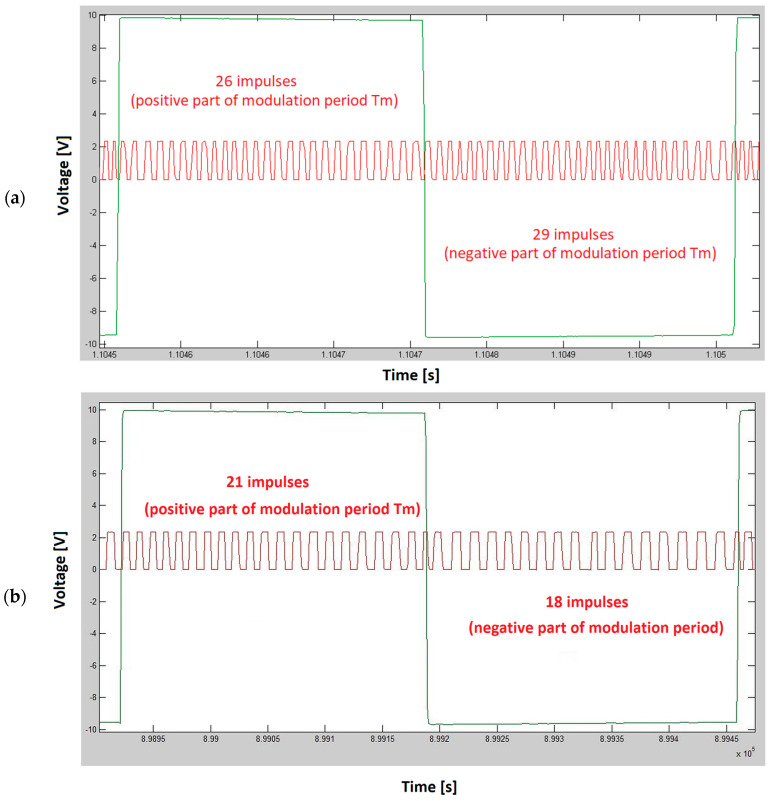
The effect of the Doppler frequency shift will cause the difference in the number of impulses during the one modulation period of the radio altimeter difference signal. In scenario (**a**) the car was moving away from the depolarization panel. In the negative half period, there will be three more impulses than in the positive half period of the difference signal. In scenario (**b**) the car was moving towards the depolarization panel. The red course represents the difference frequency signal, and the green course represents the modulation signal.

After simulations of dynamic height changes using the car, it was necessary to analyze the measured data and reliably prove the presence of the Doppler frequency. The measured data were evaluated using the Matlab environment, for which a set of commands was designed (script), which divided the data of the measured difference frequency in the rhythm of the modulation frequency into positive and negative half-periods. Then we counted the number of impulses within the positive and negative half-periods, and finally evaluated whether this number of impulses within one period of the modulation signal was the same or different. This is because the Doppler shift will cause a difference in the number of impulses when comparing the positive and negative half of one modulation period (Figure 10). When there is a positive Doppler frequency shift, the number of impulses will be higher in the positive half of the modulation period. When moving away from the depolarization panel, the Doppler shift will cause negative frequency, so the number of impulses in the first half of the modulation period will be lower than the number of impulses in the negative half period of the modulation signal [26,27,28].

**Figure 11:**
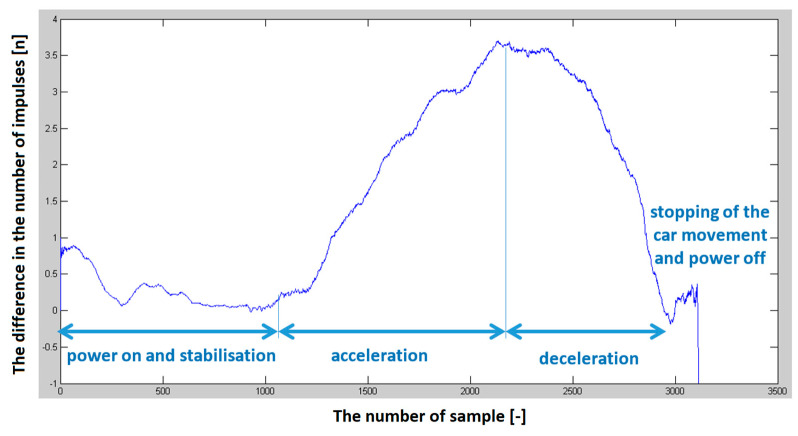
The difference in the number of impulses depends on the speed of the vehicle (car) when moving away from the depolarization panel (obstacle).

**Figure 12:**
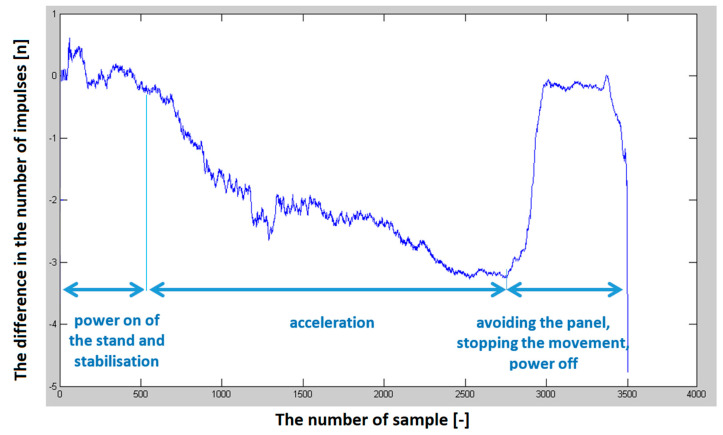
The difference in the number of impulses in the positive and negative half of the modulating period depends on the vehicle’s speed when moving towards the depolarization panel (obstacle).

Several sets of measurements were performed with different speeds of movement of the car and alternating directions of movement (heading to the depolarization panel or moving away from the depolarization panel). To evaluate the presence of this imbalance in the number of impulses in half periods representing the modulating signal, we have decided to plot this difference in the following graphs to see if there will be an observable dependency, which could also be quantified. The resulting impulse difference from control measurements was plotted in the following graphs (Figure 11 and Figure 12).

The program’s final step was to plot the graphical dependence of the difference in the number of impulses over time. The previous graphs (Figure 11 and Figure 12), showed that the hypothesis was fulfilled. Therefore, with the increasing speed of the approach of the passenger car equipped with a radio altimeter towards the imaginary obstacle, the negative difference in the number of impulses increased proportionally to the speed. In addition, when the vehicle moved in the opposite direction from the panel, this difference in the number of impulses moved from the negative to the positive region. Consequently, the polarity of the difference in the number of impulses determines whether we are approaching or moving away from the obstacle [29].

## 4. Implementation

Based on the results of previous experiments, we concluded that the simplest way of evaluating the occurrence of the Doppler frequency in radio altimeter circuits during a dynamic height change is to count the change in the number of impulses within the positive and negative half-period during one period of the modulation signal. This evaluation required creating a circuit that would divide the difference signal into sections of positive and negative half-periods in the rhythm of the modulation frequency. Subsequently, it would read the number of impulses in the thus-divided signal with two independent counters. The available technical solution used a programmable board equipped with a processor, converters, and various additional circuits—that is, a microcontroller.

Considering the available technical possibilities and our experience, the mbed Microcontroller (NXP LPC 17680) was chosen. This microcontroller uses an ARM Cortex M3 microprocessor operating at a clock frequency of 96 MHz. It has 512 KB of flash memory and 64 KB of RAM. It communicates with the environment using several buses, namely Ethernet, CAN, SPI, I2C or the USB. In addition, it contains a built-in A/D converter with a resolution of 12 bits.

**Figure 13:**
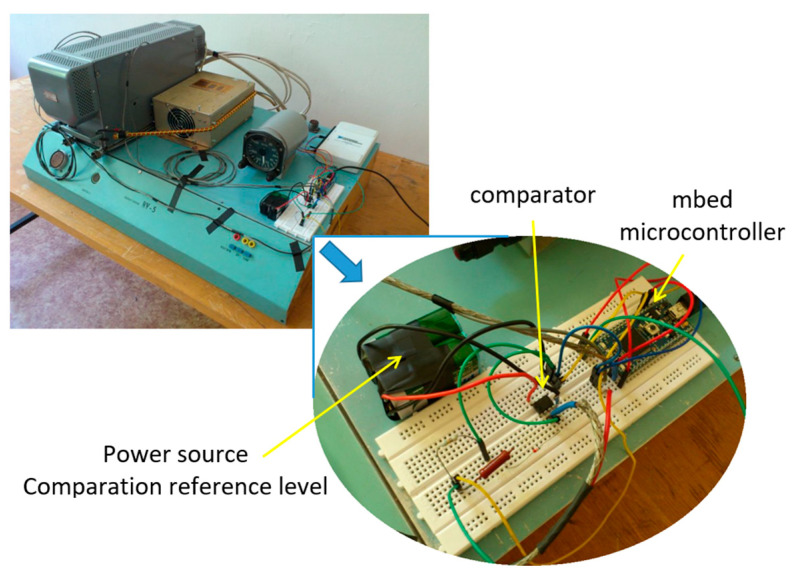
Illustration of the RV-5 experimental stand with additional circuits for evaluating the Doppler shift in difference frequency.

The disadvantages of using a microcontroller include the necessary limitation of the level of input signals, which cannot exceed the level of 3.3 Volts; otherwise, the microcontroller circuits would be damaged. Moreover, the microcontroller with an integrated counter cannot process a negative polarity signal, so it was necessary to adjust the measuring chain, including the RV-5 stand (Figure 13).

Reducing the level of the difference and modulation signal so that it does not exceed the amplitude of +3.3 V could be achieved simply by using a resistor divider with Zener diode. The difference signal changes its amplitude in the range of 0 V to +18 V; therefore, with the help of a resistor divider, this amplitude was reduced so that it did not exceed +2.8 V.

In the case of the modulating signal, the adjustment required several steps. First, the modulation signal was fed to the LM358N operational amplifier, where the input voltage was compared to zero potential. The output from the comparator was fed to a resistive divider so that the amplitude of the resulting signal did not exceed the range from 0 V to +3 V, and then this output was grounded via a semiconductor diode. This ensured that a negative polarity signal did not reach the microcontroller’s input. Subsequently, a program was designed, the task of which, similarly to the Matlab script, was to divide the difference signal in the rhythm of the modulation frequency into positive and negative half-periods, count the number of impulses within the positive and negative half-periods, and then subtract the number of impulses in the positive half-period from the number of impulses.

**Figure 14:**
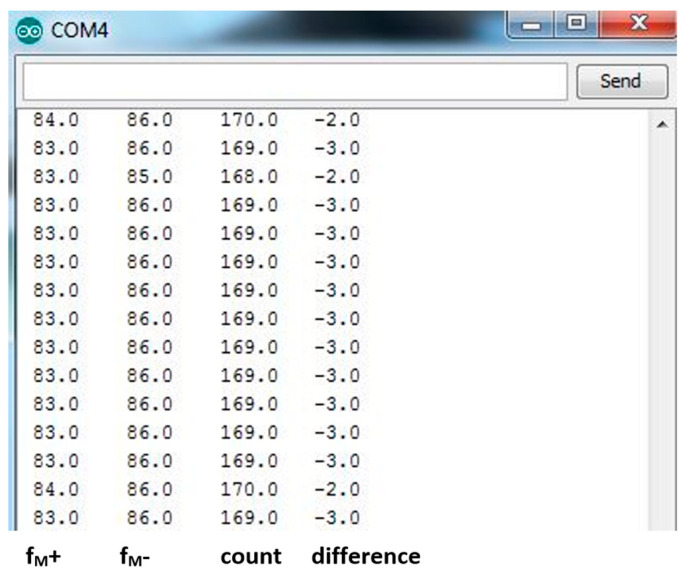
Output from the mbed microcontroller was observed by the serial monitor program.

Data extraction from the microcontroller was performed using the serial monitor program (Figure 14) with an integrated serial monitor. The first column (from the left) indicates the number of impulses in the positive half-period. The second column indicates the number of impulses in the negative half-period. The third column shows the sum of the number of impulses in the positive and negative half-periods, and the last, fourth column shows the difference in the number of impulses.

**Figure 15:**
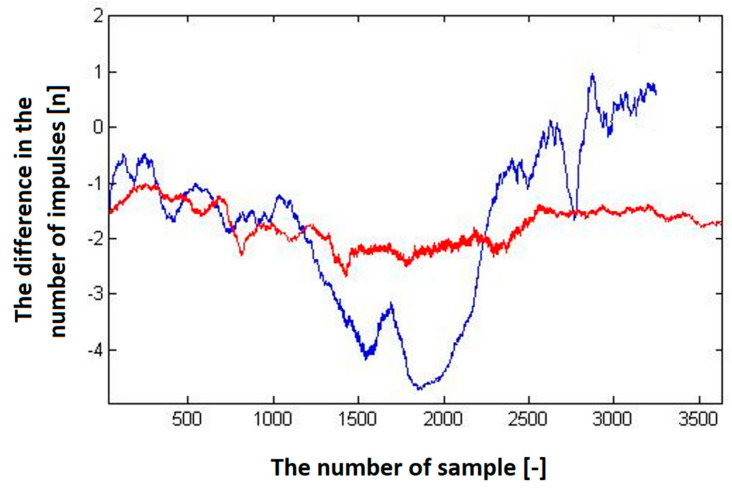
Graphic presentation of the dependence of the difference in the number of impulses over the time. The red course represents the constant speed of the vehicle approaching the panel; the blue curve represents the acceleration towards the panel.

After testing the microcontroller and a series of programs, another verification measurement was carried out using the car. An approach towards the panel was performed at a constant low speed of approximately 30 kmh^−1^. This verified the functionality of the entire connection and data collection system, and at the same time, reference data were measured for comparison. Subsequently, the measurement was performed in full acceleration mode, avoiding the panel, and then stopping. The measured data’s graphic evaluation is in Figure 15.

After this verification, the function of the microcontroller was expanded to include signaling. First, only a simple function was added, which monitored the difference in the number of impulses. If this difference exceeds a preset value in three consecutive cases, the reading will stop, and the inscription “CAUTION” will be displayed on the screen. The program modified this way was loaded into the microcontroller, and a control measurement was carried out with a passenger car. After the successfully performed measurement, when the signaling functioned as expected, it was necessary to move on to the next step, which was to activate the chosen pin of the microcontroller when the given condition was met so that, for example, it lit up the LED diode (signal bulb) for a defined time. This modification was again tested using the car. Through this series of adjustments and verification measurements, a simple device capable of signaling the influence of the Doppler frequency on the difference in the number of impulses within one modulation period was obtained.

However, for the design of a simple “anti-collision system”, this preparation had to be suitably modified and supplemented with other functions and conditions. This is because such a system would signal any height change in the terrain below the aircraft/helicopter/UAV. It was necessary to consider defining a condition that would clearly define the flight height changes, and at the same time, a difference in the number of impulses within one modulation period arises due to the Doppler effect.

The solution was to create a ratio between the sum of the impulses and the difference in the number of impulses. The sum of the number of impulses represents information about the actual flight height. And the difference in the number of impulses about the presence and size of the Doppler frequency. This ratio determines the time when the actual flight height will be zero, i.e., an imaginary impact (loss of radio altitude) with a terrain obstacle will occur. This condition was subsequently incorporated into the microcontroller program.

Another condition incorporated into the program was the setting of the so-called dead band, i.e., the program monitored the total number of impulses and stored this value in the cache memory. Afterwards, it compared this value with the reference value, which was set to the level of 160 impulses, while this number of impulses corresponds to a difference frequency of 50 kHz. This frequency corresponds to a height of 100 m. This condition established that the circuit will not signal danger if the measured height is above 100 m. Suppose the number of impulses falls below the preset value. In that case, the Doppler frequency monitoring circuit is activated—a difference in the number of impulses within the half-period of the modulation signal is created. This difference in the number of impulses is then proportional to the number of all impulses.

After defining the mentioned conditions in the program and then uploading the program to the microcontroller, the test measurement with the car was started according to the proven scenario. After a thorough analysis of the experiment scenario, the approach speeds to the depolarization panel were determined at 30 kmh^−1^, 40 kmh^−1^, 60 kmh^−1^, 70 kmh^−1^, 80 kmh^−1^ and 90 kmh^−1^. Based on previously conditions and previously measured data, the ratio of the number of all impulses to the number of differential impulses was set in the microcontroller program. Therefore, the signaling is triggered when the height falls below 100 m and is triggered if the difference in the number of impulses is more than 4. The trigger was set to start signalization of danger 3 to 5 s before the radio altitude loss (simulated impact with the obstacle).

The first test was carried out at a speed of 30 kmh^−1^ when the correctness of the connection and assembly of the entire measuring assembly and the stand were verified. It was monitored whether the radio altimeter would correctly display the simulated height during the entire route, whether the data recording would function reliably and whether false signaling would not occur.

**Figure 16:**
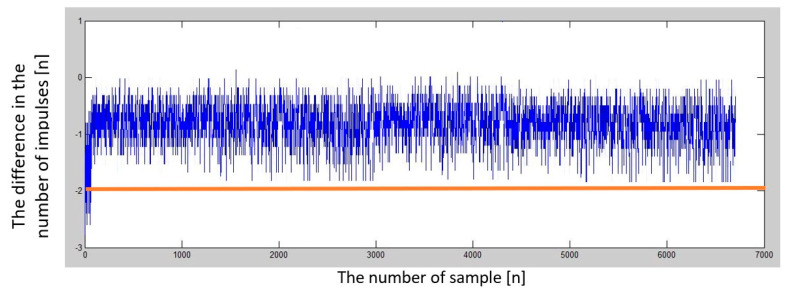
The difference in the number of impulses in time at the speed of movement 30 kmh^−1^.

The second test was carried out at a speed of 30 kmh^−1^, the difference in the number of impulses was constant with the value of −1 impulses (Figure 16).

**Figure 17:**
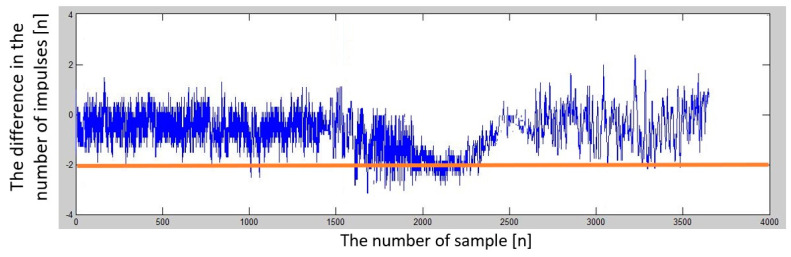
The difference in the number of impulses in time at the speed of movement 40 kmh^−1^.

**Figure 18:**
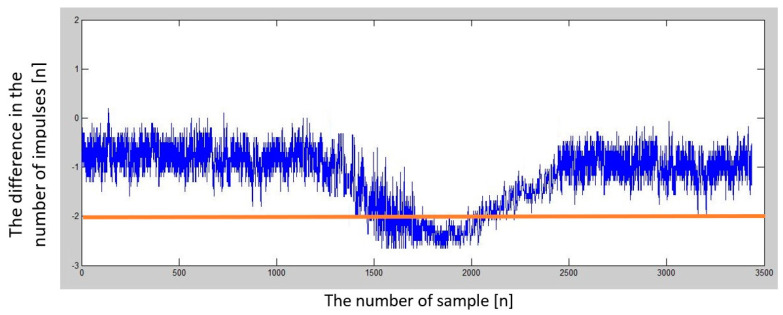
The difference in the number of impulses in time at the speed of movement 60 kmh^−1^.

At an approach speed of 40 kmh^−1^, the difference in the number of impulses began to reach the value of −2 impulses (Figure 17). At an approach speed of 60 kmh^−1^, the difference in the number of impulses began to exceed the value of −2 impulses (Figure 18). At a speed of 70 kmh^−1^, the difference in the number of impulses already exceeded the limit of the difference of −3 impulses and was approaching the limit of the difference of −4 impulses. From the progress so far, the dependence of the change in the number of impulses due to the Doppler effect on the speed of approaching the panel is linear. We have also performed a set of measurements (10 times at each approach speed) to evaluate the results and the method. The average value of the difference in the number of impulses is shown in Table 1.

After a series of previous attempts, the final phase of the experiment was reached, when signaling was expected to start. At a speed of 85 kmh^−1^, the signaling turned on approximately 70 meters from the depolarization panel and lasted less than 3 seconds, precisely during the time when the microcontroller evaluated that the predetermined conditions were met.

**Figure 19:**
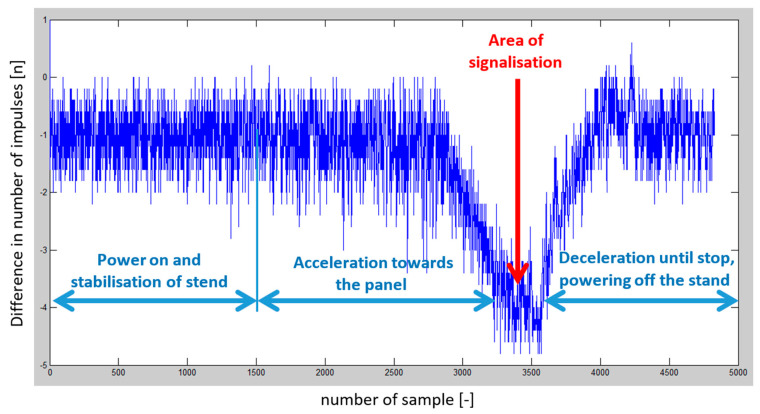
The graphic evaluation of difference of number of impulses during the final experiment.

The graphic evaluation (Figure 19) shows that the difference in the number of impulses exceeded the set limit of at least −4 impulses and even approached the limit of a difference of up to −5 impulses.

During experiments, the question of which speed range of movement of the aircraft/helicopter/UAV this method will be meaningful and purposeful for was raised. The entire consideration will be based on Table 2. Each measured value of the speed of a moving vehicle can be assigned the number of evaluated impulses “N” of the difference frequency “*f_r_*” in both half-periods of the modulation frequency *f_M_*, (column e). The speed of the vehicle moving along the road in [km·h^−1^] (column c) represents, in our case, the vertical component of the speed of the flying vehicle over the terrain. The measured value of the vehicle speed [km·h^−1^] is therefore converted to the vertical component of the speed in [m·s^−1^] (column d). Then, using relationship (20), each speed (column d) can be converted to a Doppler frequency value (column f).

Each vertical velocity component created by an approaching or receding terrain relief can be assigned the value of the cruising speed of an imaginary vehicle flying in a horizontal plane. However, the speed’s vertical component is determined by the aircraft/helicopter/UAV’s horizontal cruising speed and the steepness of the terrain obstacle (hill). From Equations (18) and (19), the ratio between the aircraft’s/helicopter’s/UAV‘s forward speed and the vertical component of the speed when flying uphill can be determined. The steepness of the relief of this hill represents a statistically optimal shape; therefore, according to the value of the angle of attack “tanβ = 0.74”, all values of the cruise speed (column b) of Table 2 were recalculated. By experimental measurement and calculation, the values in rows 3 to 8 of the given table were defined. Because the dependence of the Doppler frequency generation is linear, the remaining values of lines 1–3 and 9–18 in Table 2 were defined only by calculation.

From Table 2, it is evident that the smallest cruising speed (54 kmh^−1^) of the aircraft over the terrain that defines the vertical component of the speed (11.11 ms^−1^) at which an additional measurable Doppler frequency (326 Hz) is created, is defined in line 4 (Table 2). Adequate values measured and calculated from an experiment on a real helicopter flying over real terrain are given in line 11 (Table 2).

The analyzed scenarios and radio altimeter principle is considered for small aircraft flying with a minimum speed of approximately 100 km·h^−1^. In this case, the method should work reliably.

## 5. Conclusions

Radio altimeters are nowadays used in most aircraft and helicopters to obtain information about actual altitude (real) above the terrain. This information is useful and even crucial for a group of aircraft (avionic) systems, like auto-flight and anti-collision systems. The growing market with UAVs also creates space for the use of radio altimeters in this category of aircraft.

The radio altitude measurement accuracy is usually ± 30 cm on the most commonly used devices. This altitude measurement is affected by several negative factors, some of which could be solved or affected by signal filtration or additional internal circuitry or by a change of principle of operation. One of these factors is the Doppler shift effect, which occurs when there is a dynamic change in altitude (climb or descend). This Doppler frequency shift represents only a 4% share in the total inaccuracy of measurement, so in general, this error could be omitted. However, the authors’s team had an idea that this generally known measurement error could possibly be transformed into useful information.

For the solving of the initial hypothesis, that the Doppler shift error could be a carrier of useful information, a set of experiments was performed. The initial one was the flight experiment, which demonstrated the capability of measurement of Doppler shift in difference frequency signals. According to this, we created the mathematical theory of how it affects the creation of differential frequency. Because flight experiments are expensive, we were forced to find another solution for the simulation of dynamic altitude change of the radio altimeter.

The experiment with the depolarization panel demonstrated the applicability of the method of simulating dynamic height changes using a car and simultaneously confirmed the validity of the initially established hypothesis—that the Doppler shift of radio altimeter differential signal could be used as a source of information for the potential loss of radio altitude. The differential signal of the radio altimeter demonstrably contains (in the case of a dynamic change in height) a Doppler frequency shift, which can be used after correct detection to signal an imminent collision with an obstacle. The experiment also verified the functionality of a simple additional circuit based on a microcontroller, which was able to record the presence of the Doppler effect in the circuits of the radio altimeter and trigger signaling based on preset conditions. Furthermore, the microcontroller did not issue incorrect (false) warnings during experiments.

By adding a relatively small circuit to the original radio altimeter, without significant intervention in its design, its original functions can be significantly expanded with a new function. This predictive warning warns the crew during the flight of the danger of a possible collision with an obstacle. In this article, we showed that transforming one of the height measurement errors into a substantial advantage to increase the safety of flights and air traffic is possible. However, the method presented in this article is not final and needs to be thoroughly examined.

## Figures and Tables

**Table 1:** The average values of difference of the number of impulses obtained from the set of control measurements.

Speed of Movement of Vehicle [kmh^−1^]						
	30	40	60	70	80	90
Average value of difference of the number of impulses	1.4	2.1	3.2	3.7	4.4	4.9

**Table 2:** Measured and calculated parameters for experiment evaluation.

(a)	(b)	(c)	(d)	(e)	(f)
No.	Cruise Speed [km·h^−1^]	Vehicle Speed [km·h^−1^]	Vertical Component of Speed [m·s^−1^]	Number of Impulses [–]	Doppler Frequency [Hz]
1.	14	10	2.77	0.6	82
2.	24	20	5.55	1.0	163
3.	41	30	8.33	1.6	245
4.	54	40	11.11	2.1	326
5.	68	50	13.88	2.7	407
6.	81	60	16.66	3.3	489
7.	95	70	19.44	3.8	570
8.	108	80	22.22	4.4	652
9.	122	90	25.00	4.9	734
10.	135	100	27.77	5.4	815
11.	149	110	30.55	6.0	896
12.	162	120	33.33	6.5	978
13.	176	130	36.11	7.0	1060
14.	198	140	38.88	7.6	1140
15.	203	150	41.66	8.1	1222
16.	216	160	44.44	8.7	1304
17.	230	170	47.22	9.2	1386
18.	243	180	50	9.8	1468

## Data Availability

Not applicable.
